# Brachydactyly with Novel BMP8A and FGFR1 Variants: A Case Report with Review of Literature

**DOI:** 10.1002/ggn2.202500015

**Published:** 2025-09-04

**Authors:** Luke Hunter, Muhammad Ilyas

**Affiliations:** ^1^ Private Practice Foot and Ankle Specialist Monroe LA 71203 USA; ^2^ Department of Biological Sciences International Islamic University Islamabad Islamabad 44000 Pakistan

**Keywords:** BMP8A, brachydactyly, FGFR1, novel missense mutation, phalangeal dysplasia

## Abstract

Bone morphogenetic proteins (BMPs) and the fibroblast growth factor receptor 1 (FGFR1) gene play essential roles in the development and maintenance of the skeletal system. Brachydactyly is a genetic condition characterized by shortened or missing bones in the hands and feet. Several types of brachydactyly have been identified, each associated with different genetic mutations. However, some cases do not fit into existing classifications, necessitating further genetic investigation. A 34‐year‐old female patient with an absent middle phalanx in the second digit of her left foot and her 13‐year‐old son, who presented with absent or malformed middle and distal phalanx in all ten toes, are evaluated. Whole Genome Sequencing (WGS) analysis identifies a missense variant (c.1073A >T; p.K358M) in the BMP8A gene and a novel missense variant (c.1787C >T, p.Ser596Phe) in the FGFR1 gene. Functional protein association network analysis demonstrates a strong association of BMP8A and FGFR1 with other brachydactyly disease‐causing genes. Given that these mutations have not been previously linked to any recognized brachydactyly subtype, they likely define a distinct genetic condition. The findings suggest a novel form of brachydactyly, which naming is proposed as brachydactyly type AB.

## Graphical abstract

The current study describes a novel type of brachydactyly, characterized by absent phalanges in the toes. Whole genome sequencing identified rare missense variants in the BMP8A and FGFR1 genes, both of which are known to play a role in bone development. Bioinformatic and protein network analysis support their involvement in bone deformities. The brachydactyly type AB required further genetic and functional studies.

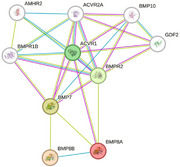

## 1 Background

Brachydactyly is a congenital hand and foot anomaly that results from underdevelopment of the phalanges, metacarpals, and/or both.^[^ ^1^ ^]^ The etiology is believed to derive from defects in blastogenesis, early embryonic development, and is a form of dysostosis where one or multiple bones are affected.^[^ ^2^ ^]^ Unlike osteochondrodysplasias, in which the entire skeleton is involved and changes may be progressive, brachydactyly is characterized by static changes present at birth.^[^ ^3^ ^]^ Interestingly, hyperphalangism and missing phalanges, which are observed features in most patients with brachydactyly, clarify its phenotypic variability.^[^ ^4^ ^]^

Brachydactyly can occur as an isolated malformation, often subtly accompanied by other anomalies, or in association with malformations such as syndactyly, polydactyly, reduction defects, or symphalangism, and is classified into types (A1 to E) and rarer forms based on the system developed by Bell and elaborated by Temtamy and McKusick, which remains a widely accepted, adaptable framework for distinguishing isolated cases, such as farabee type or stub thumb (BDD), and syndromic variants while allowing for incorporation of newly identified subtypes.^[^ ^5^ ^]^

Abnormality in skeletal structure, brachydactyly, involves one or more of the digits being short and is caused by an error in embryological development. The upper limb is divided into zones: the stylopod (humerus), zeugopod (radius and ulna), mesopod (carpal bones), and autopod (metacarpals and phalanges), of which defects in brachydactyly mainly involve the autopod, while genetic and molecular signaling regulatory the size and shape of the cartilaginous templates.^[^ ^4^ ^]^

Type A1 (BDA1), or Farabee‐type brachydactyly, results from heterozygous missense mutations in the signaling molecule *Indian Hedgehog* (*IHH*).^[^ ^6^ ^]^ While homozygous inactivation of *IHH* leads to lethal chondrodysplasia, heterozygous mutations cause hypoplastic or absent middle phalanges.^[^ ^7^ ^]^ Type B (BDB) is caused by truncating mutations in the receptor tyrosine kinase *ROR*.^[^ ^8^ ^]^ These mutations impair chondrocyte differentiation, with distal mutations leading to a more severe phenotype, including hypoplasia or aplasia of distal phalanges and nails.^[^ ^9^, ^10^ ^]^ Type C (BDC) results from heterozygous frameshift or nonsense mutations in growth/differentiation factor 5 (*GDF5)*, a cartilage‐derived morphogenetic protein critical for limb morphogenesis.^[^ ^11^ ^]^ Homozygous mutations in *GDF5* lead to more severe chondrodysplasia, such as Grebe,^[^ ^12^ ^]^ DuPan syndrome,^[^ ^13^ ^]^ and Hunter‐Thompson syndromes.^[^ ^14^ ^]^ During embryogenesis, patterning of the autopod is regulated by key molecular players such as Sonic Hedgehog (SHH), HOX genes, and fibroblast growth factors (FGFs), which guide the development of skeletal templates. These templates are populated by extracellular matrix‐rich cell aggregates, forming the skeletal analog. Endochondral ossification, the predominant mechanism for limb bone formation, is mediated by factors like SOX9, ROR2, BMPs (Bone Morphogenetic Proteins), GDF5, Indian Hedgehog (IHH), and WNT.^[^ ^15^ ^]^ Conversely, the distal tips of the phalanges undergo membranous ossification, guided by RUNX2.^[^ ^4^ ^]^ Genetic mutations underlie brachydactyly, with distinct phenotypes arising from disruptions in endochondral bone formation or skeletal patterning. For instance, mutations in GDF5, BMPR1B, IHH, and ROR2 disrupt the TGF‐β signaling cascade essential for endochondral ossification, contributing to types A1, A2, B, and C.^[^ ^4^, ^16^ ^]^ Insights from developmental biology, including the role of BMP/p‐SMAD signaling in phalangeal precursor cells, explain digit‐specific variations like the preservation of the ring finger in certain types of brachydactyly.^[^ ^4^ ^]^ These findings highlight the intricate interplay between genetic, molecular, and embryological factors in shaping limb development.

Brachydactyly is genetically linked to mutations in developmental and glycosyltransferase‐related genes (e.g., *HOXD13, GDF5, CHSY1*) affecting bone morphogenesis and glycosaminoglycan biosynthesis pathways.^[^ ^17^ ^]^ Brachydactyly arises from disruptions in genes critical for limb patterning and development during embryogenesis, including HOXD13, which regulates the spatial and temporal expression of limb structures. Mutations in HOXD13, critical for autopod patterning and elongation, lead to types A4, D, and E. The severity of brachydactyly correlates with the degree of HOXD13 expression, as lower expression levels yield shorter bones. Mutations in HOXD13, such as polyalanine expansions, frame shifts, or missense mutations, can impair its function as a homeodomain transcription factor, affecting its DNA‐binding affinity and altering gene networks responsible for bone morphogenesis. These genetic disruptions lead to specific phenotypes, such as brachydactyly types D and E, characterized by shortened metacarpals, metatarsals, and phalanges.^[^ ^18^ ^]^

Fibroblast Growth Factors (FGFs) play a critical role in regulating the formation and number of phalanges in developing digits through their interaction with the Apical Ectodermal Ridge (AER).^[^ ^19^ ^]^ FGFs are controlled by Bone Morphogenetic Proteins (BMPs) and their antagonist GREMLIN1, creating a dynamic feedback loop essential for skeletal patterning.^[^ ^19^ ^]^ BMP activity, which varies across interdigital regions, establishes unique phosphorylated‐SMAD (p‐SMAD) activity signatures that influence digit formation. In the chick model, lower BMP/p‐SMAD activity correlates with fewer phalanges, as seen in digit 1, while higher activity corresponds to more phalanges, as seen in digit 3. Dysregulation of FGFs, BMPs, or GREMLIN1 activity can result in skeletal anomalies such as hyperphalangism or missing phalanges, both common features of brachydactyly.^[^ ^4^ ^]^

Brachydactyly type A1 (BDA1) primarily affects the shortening of middle phalanges in both the fingers and toes due to missense mutations in the *Indian hedgehog* (*Ihh*) gene. These mutations result in a gain‐of‐function effect that disrupts the normal processes of chondrocyte differentiation, joint formation, and bone development.^[^ ^20^ ^]^ In toes, this manifests as shortened middle phalanges similar to the changes seen in fingers.^[^ ^20^ ^]^ The *Ihh* pathway, essential for regulating endochondral ossification, is altered by these mutations, impairing the interaction of *Ihh* with its inhibitors, such as Hip and *Ptch*.^[^ ^21^ ^]^ This leads to abnormal signaling, which affects the growth and structure of phalanges in both digits and toes. Mouse models carrying similar mutations show disrupted *Ihh* signaling, with ectopic *Shh* expression further complicating chondrocyte differentiation and joint formation.^[^ ^22^ ^]^ The involvement of toes in BDA1 underscores the systemic nature of the disruption in *Ihh* signaling across the appendicular skeleton.

Brachydactyly type A2 (BDA2) is marked by shortened or absent middle phalanges of the second and fifth fingers, with the index finger often deviated medially due to an abnormally shaped middle phalanx.^[^ ^20^ ^]^ In the toes, BDA2 manifests as short, broad, laterally deviated big toes (halluces) and medially deviated second toes.^[^ ^20^ ^]^ This condition may overlap with brachydactyly type C (BDC), showing phenotypic variability even within families.^[^ ^20^ ^]^ BDA2 is genetically heterogeneous, caused by mutations in *GDF5* and *BMPR1B*, which are crucial for BMP signaling.^[^ ^20^ ^]^ Specific *GDF5* mutations impair binding to BMPR1B, while dominant‐negative mutations in *BMPR1B* itself and duplications in the regulatory region of *BMP2* can also lead to BDA2.^[^ ^23^, ^24^ ^]^ Mutations in the *GDF* inhibitor *NOG* or activating *GDF5* mutations result in proximal symphalangism (SYM1). A novel *BMPR1B* mutation, R486Q, causes either BDA2 or a BDC/SYM1‐like phenotype.^[^ ^25^ ^]^ Functional studies show that the R486Q mutation strongly inhibits chondrogenesis and suppresses SMAD‐dependent signaling, with more severe effects than the R486W mutation. This highlights how disruptions in the NOG‐GDF5‐BMPR1B pathway lead to overlapping phenotypes, dependent on the mutation's quantitative impact and specific mechanisms.^[^ ^25^ ^]^ These genetic changes disrupt BMP signaling, shifting activity from BMPR1B to BMPR1A, and altering the development of digits and toes. Regulatory mutations in BMP pathways underscore the complexity of signaling required for proper skeletal formation.

Brachydactyly type D (BDD) is characterized by a shortened distal thumb phalanx and broad toes, with complete penetrance in females and incomplete penetrance in males.^[^ ^20^ ^]^ Bilateral expression occurs in ≈75% of affected individuals.^[^ ^20^ ^]^ A mutation in *HOXD13* has been implicated in BDD.^[^ ^18^ ^]^ Grebe, Hunter‐Thompson, and Du Pan acromesomelic dysplasias are severe autosomal recessive limb disorders caused by homozygous *GDF5* mutations.^[^ ^20^ ^]^ Grebe dysplasia exhibits profound limb shortening, absent ossification of phalanges, and rudimentary digits resembling toes. Hunter‐Thompson dysplasia presents similarly but less severely, while Du Pan Dysplasia involves fibular hypoplasia and mild brachydactyly overlapping BDA1, BDA2, and BDC. Feingold syndrome, caused by *NMYC* mutations–a member of the MYC family of oncogenes, is characterized by syndactyly of toes 2–3 and 4–5, hypoplastic middle phalanges, and other systemic abnormalities.^[^ ^26^, ^27^ ^]^ Conditional *NMYC* inactivation in mice results in skeletal and joint abnormalities, implicating *NMYC* in interdigital cell death and joint formation, linking its dysfunction to syndactyly and brachydactyly.^[^ ^28^ ^]^

## 2 Case Presentation

### 2.1 Clinical Details

#### 2.1.1 Patient 1

**Figure 1:**
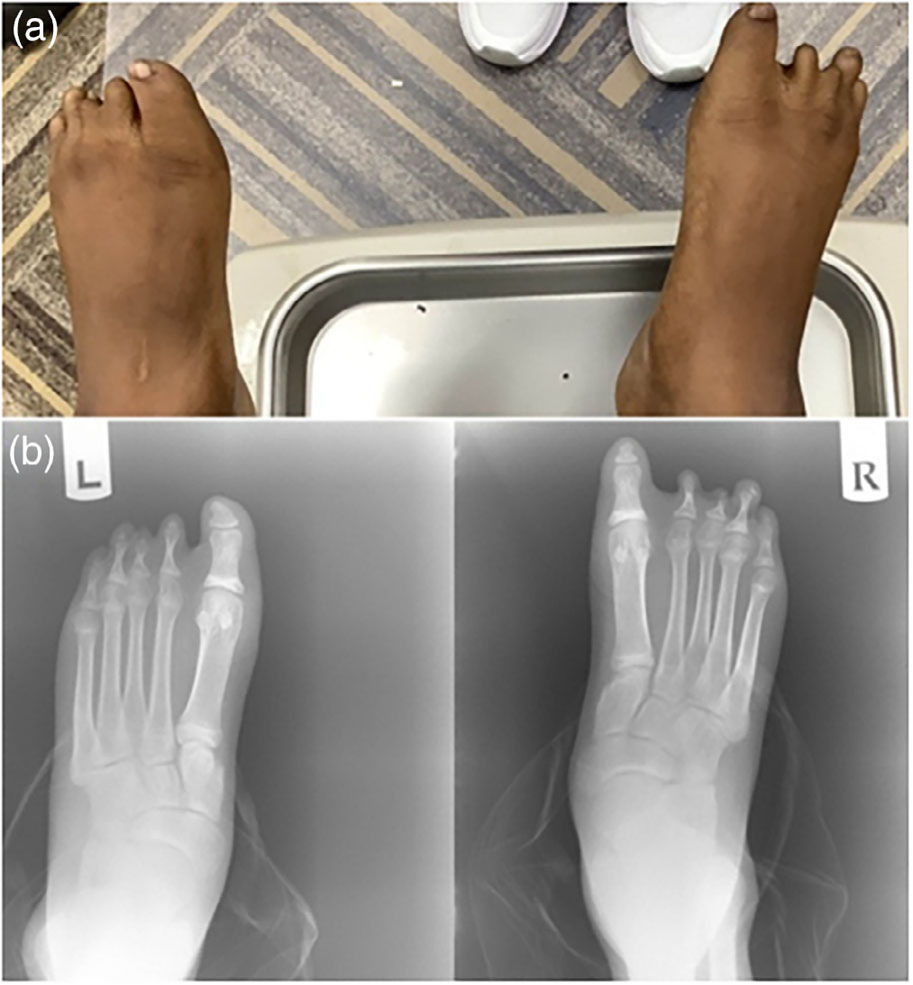
A) Photo of the boy's left and right feet at 13 years of age; digital malformations are clearly shown in the picture. B) Both foot radiographs of the boy at 13 years of age show absent or malformed phalanges.

The male proband was born as a premature baby at 30 weeks of gestation. There was no unusual exposure during pregnancy, and the parents were non‐consanguineous. At birth, he was noted to have congenital anomalies of the feet. He started walking at the age of 2 years. Throughout childhood, the patient has had vocal issues and some bowel/bladder issues. At 13 years old, he was brought into the clinic with a primarily cosmetic complaint of having toe deformities. X‐ray examination revealed he had bony deformities in all 10 of his toes, ranging from phalangeal aplasia to phalangeal hypoplasia (**Figure** **1**). This clinical presentation of all 10 toes is similar to type B brachydactyly, which is one of the rarer types and which typically affects the fingers instead of the toes.

#### 2.1.2 Patient 2

**Figure 2:**
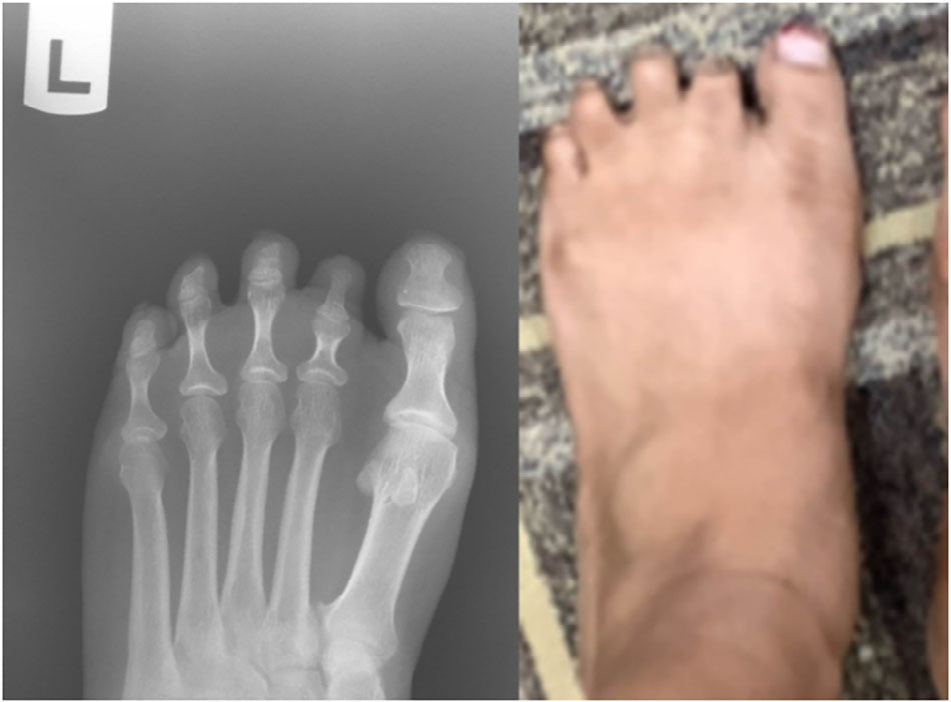
Imaging of a 34‐year‐old female exhibiting digit deformity in her left foot, shown in the radiograph and photograph.

A 34‐year‐old female with no significant medical history possesses a left 2nd toe deformity, shown in **Figure** **2**. She reported normal birth and development. This clinical presentation of an isolated absent middle phalanx is similar to type A brachydactyly (Figure 2).

### 2.2 Whole Genome Analysis

To identify the disease‐causing gene(s) and variant(s) in the family, mother and son samples were sent for whole genome sequencing to Nebula Genomics. Sequencing results were analyzed online by using the gene analysis tool gene.iobio v4.10 powered by Nebula genome. After initial data filtration, we pinpointed two genes containing pathogenic variants. The BMP8A gene, located on chromosome 1, containing the missense variant (c.1073A >T, p.lys358met) and rs1194767366 located in exon 7, is the causal variant underlying the missing first metatarsals and middle phalanges on any toes in this isolated family based on bioinformatic pathogenicity and conservation score. IGV views of the BMP8A and FGFR1 gene mutations of the mother's sample are shown in Figures S1 and S2 (Supporting Information) for the mother and Figures S3 and S4 (Supporting Information) for the son, respectively. IGV view of the BMP8A and FGFR1 gene mutation of the mother sample can also be accessed on Harvard Dataverse by using the link https://dataverse.harvard.edu/dataset.xhtml?persistentId=doi:10.7910/DVN/YKRHLF. IGV view of the son's sample was available on Harvard dataverse https://dataverse.harvard.edu/dataset.xhtml?persistentId=doi:10.7910/DVN/NUNW1H. Both sequenced subjects carried a heterozygous allele (A/T) at rs1194767366. Conservation is a very important criterion for measuring the significance of a variant on an evolutionary scale. The combined annotation dependent depeletion (CADD) score for this variant (rs1194767366) is 25, considered a damaging derangement for the genome, and the genomic evolutionary rate profiling GERP++ score of 4.4 is considered extremely conserved. This variant is considered pathogenic based on several prediction tools, including Polyphen score,^[^ ^1^ ^]^ damaging, and rare exome varaint ensemble learner (REVEL) score is (0.71) pathogenic.

The second gene is the FGFR1 gene, located on chromosome 8, containing the missense variant (c.1787C >T, p.Ser596Phe), rs1463369542, located in exon 13. The FGFR1 gene plays a crucial role in bone development and homeostasis; a mutation in the FGFR1 gene most likely caused the disease in the patient based on bioinformatic pathogenicity and conservation score. Both sequenced subjects carried a heterozygous allele (C/T) at rs1463369542. This is a very rare variant and has not been presented in the gnomAD database. Conservation is a very important criterion for measuring the significance of a variant on an evolutionary scale. The CADD score for this variant (rs1463369542) is 29, considered a damaging derangement for the genome, and the GERP++ score of 5.4 is considered extremely conserved. This variant is considered pathogenic based on several prediction tools, including Polyphen score^[^ ^1^ ^]^ damaging, REVEL (0.82) pathogenic, SIFT deleterious (0), and DEOGEN2 pathogenic (0.92). These prediction tools score backing the contention that the amino acid changes caused by both variants are causing functional changes in the gene's protein.

### 2.3 Management and Treatment

As with most pedal presentations of brachydactyly, due to the asymptomatic presentation in both patients, treatment options were primarily conservative, with an emphasis on how to properly fit shoes and offer custom orthotics to possibly improve the patients’ biomechanics.

Rare surgical interventions for brachydactyly are primarily indicated for brachydactyly phenotypes affecting the hands. Bone grafting or bone lengthening procedures are more indicated for long bone involvement, like the metacarpals. Should the digits become symptomatic in the future due to the malformation of the interphalangeal joints, surgical options would likely be limited to a possible arthrodesis, but more likely a partial or total amputation of the affected digit?

## 3 Discussion and Conclusion

Although there are several different types of brachydactyly cases reported in the literature, this is the first time we've had a brachydactyly presentation, but the absence of the known mutations indicates this is a novel type of brachydactyly.

**Figure 3:**
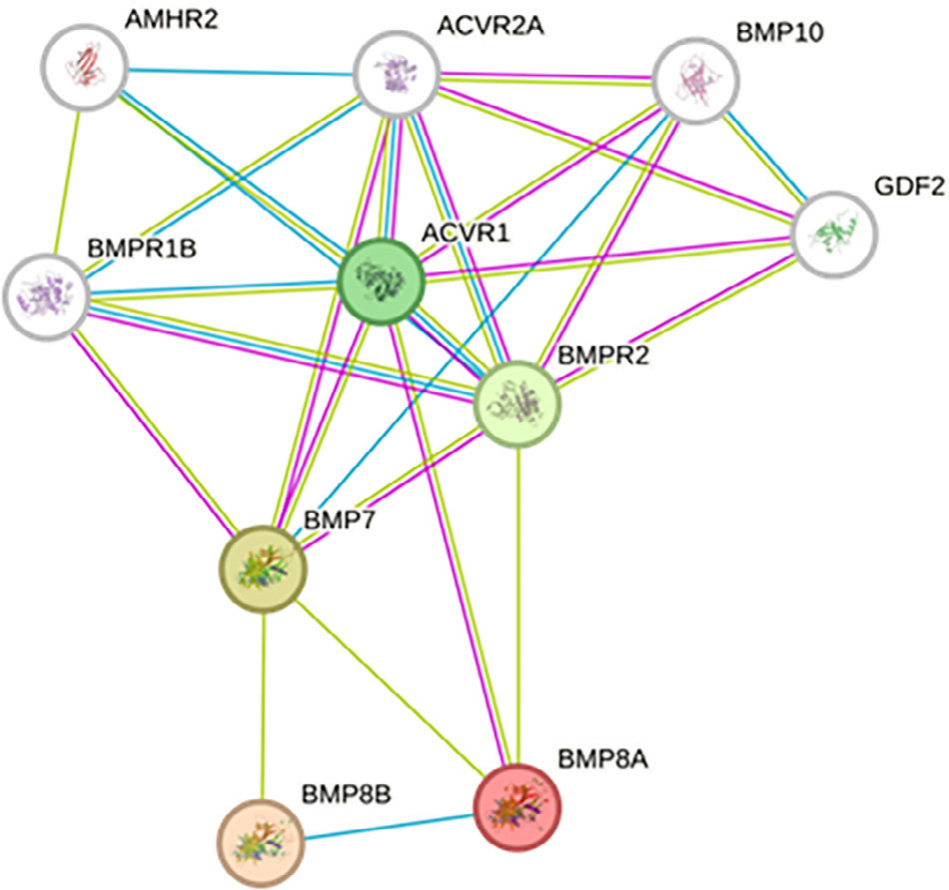
BMP8A and BMP8B show protein homology, and BMP8A shows strong interaction and fusion with other genes, causing bone‐related disorders.

We propose the classification of a new type of brachydactyly and name it “brachydactyly type AB.” This is the first report that describes brachydactyly AB, in which the mother and son present with different phenotypes of brachydactyly. The novel missense variant c.1073A >T, p.Lys358Met in the BMP8A gene and the novel missense variant c.1787C >T, p.Ser596Phe in the FGFR1 gene in both samples are not associated with other forms of brachydactyly. The FGFR1 gene is expressed in the cranial neural crest and functions in the embryogenesis of bones.^[^ ^28^ ^]^ The FGF signaling mechanism is important for ventral telencephalon development and digit formation at the time of embryogenesis.^[^ ^29^ ^]^ So far, 297 disease‐causing variants have been reported in the FGFR1 gene according to the HGMD database. Variants in the FGFR1 gene cause different diseases, including hand and foot malformation.^[^ ^30^ ^]^ Bone morphogenetic proteins are a part of cell signaling molecules that belong to the Transforming Growth Factors‐β (TGF‐β) family of proteins. BMPs induce bone formation, embryogenesis, development, and tissue homeostasis. Impaired BMP signaling is associated with bone deformities mentioned in **Table** **1**.^[^ ^31^ ^]^ Different studies confirmed the ability of BMPs to induce mesenchymal stem cells to differentiate into bone, endorsing their role in cartilage, bone formation, and osteogenesis.^[^ ^32^, ^33^ ^]^ We firmly believe that our reported mutations in the FGFR1 and BMP8A genes are responsible for the disease in the mother and son. Similarly, the STRING network (**Figure** **3**) shows strong interaction of the BMP8A gene with other similar genes, causing bone deformities in the patients.

**Table 1:** Describes more on the existing classification of brachydactyly.

Category	Subtype/Condition	Affected region	Genetic mutation
1. Middle Phalanges	A1 (Farabee type)	Middle phalanges of all digits (undeveloped or fused)	*IHH*
	A2 (Mohr‐Wreidt type)	Middle phalanges of index finger and/or 2nd toe	*BMPR1B, BMP2, GDF5*
	A3 (Brachymesophalangy V)	Middle phalanx of the 5th (little) finger	*HOXD13*
	A4 (Temtamy type)	Middle phalanges of the 2nd and 5th digits	*HOXD13*
	A5	Middle phalanges of digits 2–5, associated with nail dysplasia	Not specified
2. Distal Phalanges	B1	Distal phalanges of digits 2–5; nail aplasia; middle phalanges may be hypoplastic	*ROR2*
	B2	Not specified	*NOG*
	Kirner deformity (Dystelephalangy)	Distal phalanx of the 5th finger; radial bowing	Not specified
	D (Stub Thumb)	Distal phalanx of 1st digit (thumb)	*HOXD13*
3. Metacarpals and Metatarsals	E	Metacarpals and metatarsals	*HOXD13*
	Brachymetatarsus IV	Fourth metatarsal, unilateral or bilateral shortening	Not specified
4. Combined Phalangeal and Hyperphalangism	C (Haws type)	Brachymesophalangy of digits 2, 3, 5; hyperphalangy of 2nd, 3rd; shortening of 1st metacarpal	*GDF5, HOXD13*
5. Syndromic Forms	Sugarman Brachydactyly	Hallux (proximal and dorsal displacement)	Not specified

Moreover, the mother and son whole genome analysis did not have mutations in the IHH and BDA1B genes (Type A1), BMPR1B and GDF5 (Type 2), HOXD13 (Type A3 and A4), GDF5 (Type C), HOXD13 (Type B and Type E), or HOXD13 (Type B and E combined, Ballard syndrome or Pitt‐williams). These mentioned types are responsible for different types of brachydactyly.

The current study has a few limitations. First, it is based on only two related individuals, a mother and son, and no other family members have any clinical presentation of brachydactyly, nor were they available to participate. Whole‐genome analysis was performed on both individuals, and data analysis was conducted using an online platform provided by Nebula Genomics. The pathogenicity of the selected variants was assessed only using computational tools such as the CADD score, REVEL score, and GERP score. The absence of functional validation studies represents another limitation that should be addressed in future research. Further studies involving larger sample sizes are needed to validate these findings, although it is noteworthy that the identified variants were not present in population databases such as the gnomAD database. More in‐depth studies in the future will enhance our understanding of the molecular mechanisms underlying brachydactyly Type AB.

## Conflict of Interest

The authors declare no conflict of interest.

## Author Contributions

The authors confirmed their contribution to the case report as follows. L.H. performed the clinical analysis and diagnosis of the patients, conceptualized the study, co‐wrote the manuscript, obtained funding, and performed final proofreading and editing. M.I. performed genomic analysis, bioinformatic analysis, co‐wrote the manuscript, designed the case report, and performed editing.

## Ethical Approval and Consent to Participate

The study is approved by the Institutional Ethical Review Committee of the Department of Biological Science, International Islamic University Islamabad, Pakistan. The research consent form was obtained from the research participant.

## Peer Review

The peer review history for this article is available in the Supporting Information for this article.

## Supporting information



Supporting Information

Supplementary Information: Record of Transparent Peer Review

## Data Availability

The data that support the findings of this study are openly available in Figshare database at https://doi.org/10.6084/m9.figshare.29965811.v1, https://doi.org/10.6084/m9.figshare.29966686.v2, reference number 34.
