# Supplementary material for: Brachydactyly with Novel BMP8A and FGFR1 Variants: A Case Report with Review of Literature
Source: Adv Genet (Hoboken). 2025 Sep 4;6(3):e00015. doi: 10.1002/ggn2.202500015 (PMC12482929; doi:10.1002/ggn2.202500015)
Supplement: Supplementary file 1 — Supporting Information [file GGN2-6-e00015-s002.docx]

Brachydactyly with Novel BMP8A and FGFR1 Variants: A Case Report with Review of Literature

# Supplementary figures BAM file IGV view of the mutation in BMP8A gene and FGFR1 gene mother sample.


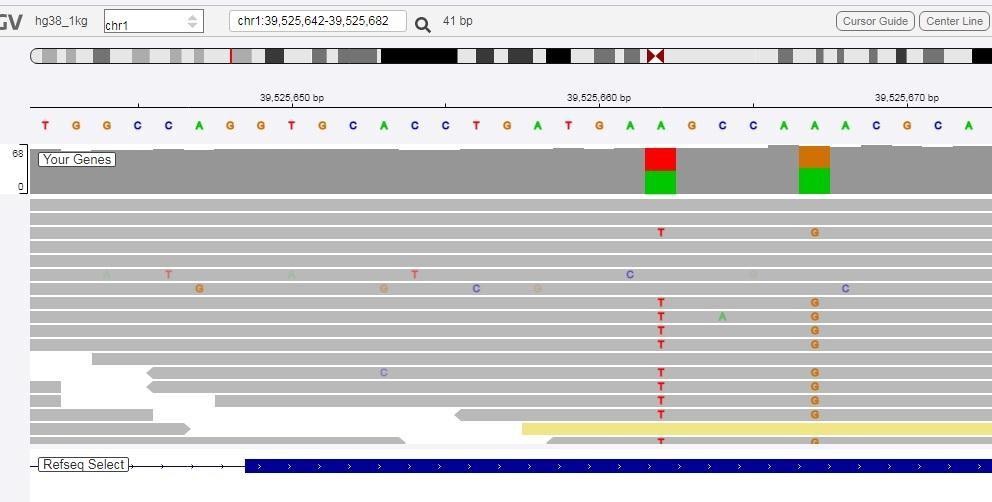


Supplementary Figure 1: Mother sample BMP8A gene located on chromosome 1 containing the missense variant (c.1073A>T, p.lys358met), rs1194767366.


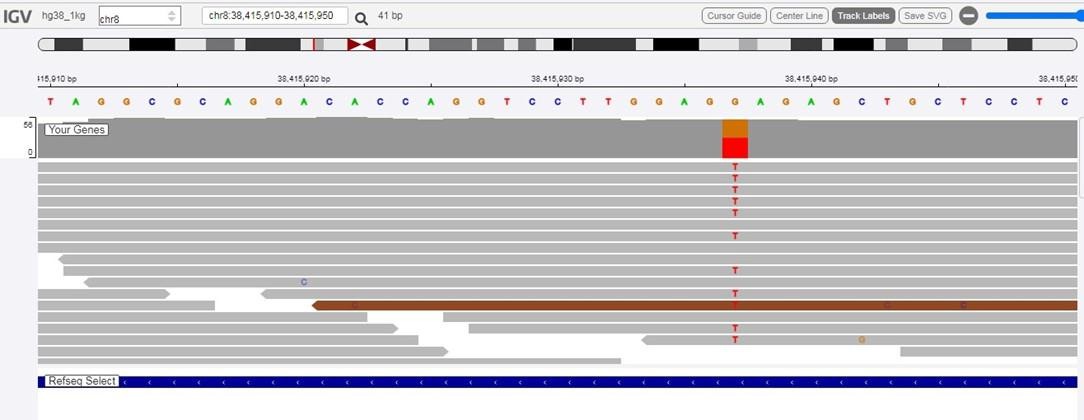


Supplementary Figure 2: IGV view of BAM file mother sample, FGFR1 gene located on chromosome 8 containing the missense variant (c.1787C>T, p.Ser596Phe), rs1463369542 located in the exon 13.

# Supplementary figures BAM file IGV view of the mutation in BMP8A gene and FGFR1 gene son sample.

**
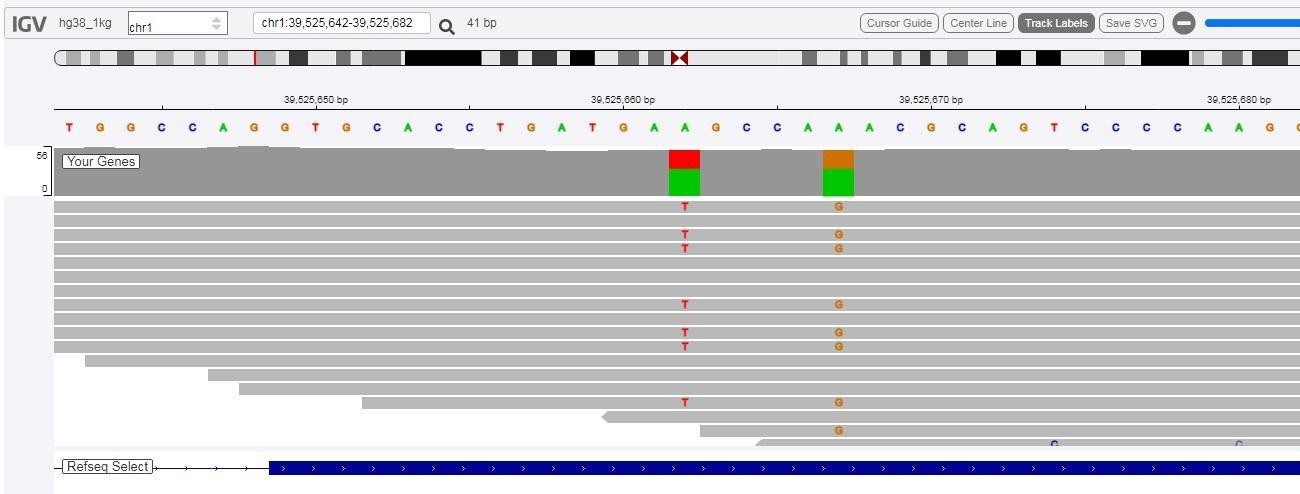
**

Supplementary Figure 3: Son sample BMP8A gene located on chromosome 1 containing the missense variant (c.1073A>T, p.lys358met), rs1194767366.


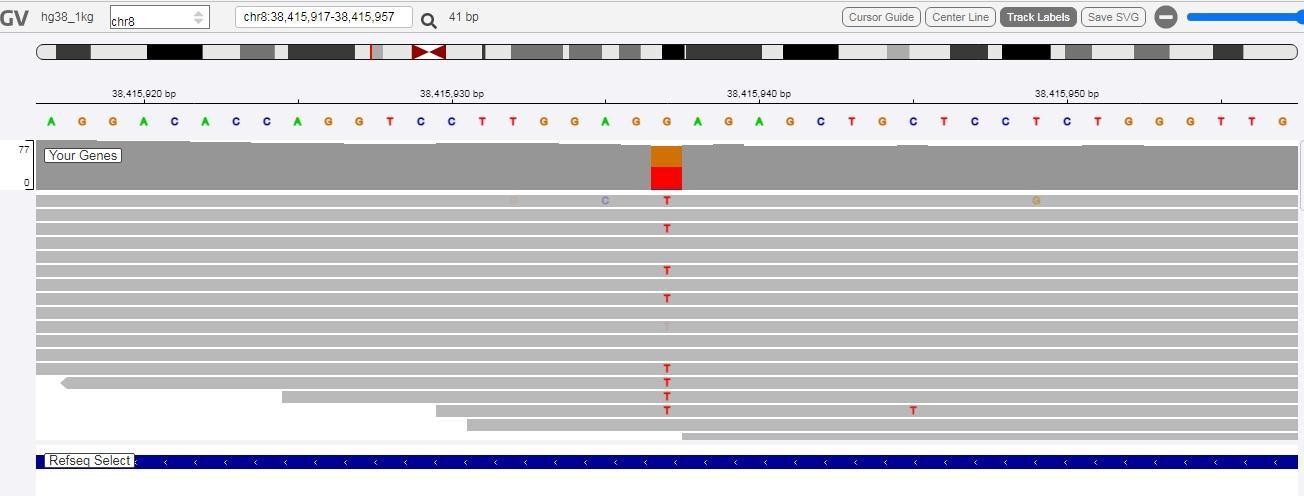


Supplementary Figure 4: IGV view of BAM file son sample, FGFR1 gene located on chromosome 8 containing the missense variant (c.1787C>T, p.Ser596Phe), rs1463369542 located in the exon 13.

The son and mother vcf file containing genomic data are accessed on Figshare database: Patient 1: <https://doi.org/10.6084/m9.figshare.29965811.v1>

Patient 2: <https://doi.org/10.6084/m9.figshare.29966686.v2>
