# Supplementary material for: Brachydactyly with Novel BMP8A and FGFR1 Variants: A Case Report with Review of Literature
Source: Adv Genet (Hoboken). 2025 Sep 4;6(3):e00015. doi: 10.1002/ggn2.202500015 (PMC12482929; doi:10.1002/ggn2.202500015)
Supplement: Supplementary file 2 — Supplementary Information: Record of Transparent Peer Review [file GGN2-6-e00015-s001.docx]

Record of Transparent Peer Review

Brachydactyly with Novel BMP8A and FGFR1 Variants: A Case Report and Review of the Literature

Data submitted: 13-Apr-2025

1st Editorial Decision: 23-Jun-2025

1^st^ Revision Received: 15-Jul-2025

2^nd^ Editorial Decision: 31-Jul-2025

Last Revision Received: 22-Aug-2025

Accepted: 24-Aug-2025

Revision with responses: 15-Jul-2025

***Reviewer #1***

**Comment 1**

The authors investigated "a novel form of brachydactyly, which we propose naming as

brachydactyly type AB" according to their recent findings.

Response:

We thank the reviewer for their careful review and constructive suggestions. As recommended, we have

changed the title from "Novel Brachydactyly Type AB Syndrome" to “Brachydactyly Type AB”.

**Comment 2**

However, to give a new name to a syndrome, functional studies should be involved.

However, the genetic information and figures are missing. The authors should show IGV

views, as well as family segregation analysis by Sanger and family pedigree.

Response:

We have also excluded the word "syndrome" from our study. All available genetic information has been

added to the manuscript. The IGV views of the genomic analyses of the mother’s and son’s samples

have been included in the supplementary files and are also published online on Harvard Dataverse (data

link provided in the manuscript). If the reviewer or editor wishes to review the genomic data in detail,

access to the Nebula Genomics account can be shared directly with them. In this family, only the

mother’s and son’s samples were sent for genomic analysis. Unfortunately, they are not cooperating for

additional testing, including the suggested Sanger sequencing and family segregation analysis.

***Reviewer #2***

**Comment 1**

While the paper gives a comprehensive background, adding more detail on how the newly

proposed brachydactyly type AB differentiates itself from other types would provide clarity.

Expanding on how exactly the identified variants in BMP8A and FGFR1 might disrupt

normal signalling pathways would enhance reader understanding.

Response Letter to Reviewers

Response:

We thank the reviewer for their valuable comments on our paper. In response to the first comment, we

would like to highlight that the existing classifications of brachydactyly are presented in tabular form in

our manuscript. Our patient’s genetic mutations and X-ray findings do not match any current

classification, which is why we proposed the term "Type AB brachydactyly", as the X-ray findings are

most similar to both Type A and Type B brachydactyly.

Furthermore, the BMP8A gene is clearly involved in bone morphogenesis (the process by which bone

tissue develops its shape and structure during embryogenesis). A pathogenic mutation in this gene can

disrupt its normal function. FGFR1 is a growth factor gene that also plays an important role in bone

morphogenesis during embryogenesis. The roles of FGFR1 and BMP8A are already discussed in the

discussion section; however, due to word limits, we are unable to add further details. We hope the

reviewer will understand our position.

**Comment 2**

The authors can include a brief section on study limitations, such as the small sample size

and reliance on computational predictions without functional validation, acknowledging

potential weaknesses and setting the stage for future research. Given that the study is based

on two related cases, discussing the need for larger cohort analyses or validation in

unrelated cases would add depth to the discussion. Addressing these aspects would enhance

the generalizability and applicability of the findings.

Response:

The current study has a few limitations. First, it is based on only two related individuals, a mother and

son, and no other family members have any clinical presentation of brachydactyly nor were available to

participate. Whole-genome analysis was performed on both individuals, and data analysis was

conducted using an online platform provided by Nebula Genomics. The pathogenicity of the selected

variants was assessed only using computational tools such as the Combined Annotation Dependent

Depletion (CADD) score, rare exome variant ensemble learner (REVEL) score, and genomic

evolutionary rate profiling (GERP) score. The absence of functional validation studies represents

another limitation that should be addressed in future research. Further studies involving larger sample

sizes are needed to validate these findings, although it is noteworthy that the identified variants were

not present in population databases such as the gnomAD database. More in-depth studies in the future

will enhance our understanding of the molecular mechanisms underlying Brachydactyly Type AB.

***Editor’s comments***

The links provided in the Data Availability Statement are currently inaccessible. Kindly update the correspondence information and submit it as supporting data in a Word file. Otherwise, could you please deposit the data in a global accessible public available database?
